# Dr. Rush's Recantation Respecting Yellow Fever

**Published:** 1806-03

**Authors:** L. J. Jardine

**Affiliations:** Liverpool


### *To the Editors of the Medical and Physical Journal.*

Gentlemen,

My friend, Dr. Rush, of Philadelphia, has, in his new edition of his Medical Inquiries and Observations, retracted his former opinion respecting the contagious nature of the yellow fever; and, being desirous of making this recantation as public as possible, he has requested me to obtain the insertion of the following extract from his Preface, in one of the periodical works of this country. If you will have the goodness to give it a place in your your valuable Journal, my friend's object will be fully accomplished, and you will much oblige,

Yours, &c.

*Liverpool, Feb.* 10, 1806. L. J. JARDINE.

---

" In the fourth volume, the Reader will find a retrac-
" tion of the Author's former opinion of the yellow fever
" spreading by contagion. He begs forgiveness of the
" friends of science and humanity, if the publication of
" that opinion has had any influence in increasing the
" misery and mortality attendant upon that disease. In-
" deed, such is the pain he feels, in recollecting that he
" ever entertained or propagated it, that it will long, and
" perhaps always, deprive him of the pleasure he might
" otherwise have derived from a review of his attempts
" to fulfil the public duties of his profession."

